# Metagenomic analysis reveals specific BTEX degrading microorganisms of a bacterial consortium

**DOI:** 10.1186/s13568-023-01541-y

**Published:** 2023-05-17

**Authors:** Hui-jun Wu, Xian-yuan Du, Wen-jing Wu, Jin Zheng, Jia-yu Song, Jia-cai Xie

**Affiliations:** 1 State Key Laboratory of Petroleum Pollution Control, National Petroleum Corporation Research Institute of Safety and Environmental Technology, 102206 Beijing, China; 2 College of Life Science, Northwest University, 710000 Xian, China

**Keywords:** Petroleum hydrocarbon, Microbial consortium, Cyclohexane biodegradation, BTEX biodegradation, Metagenomic analysis

## Abstract

Petroleum hydrocarbon contamination is of environmental and public health concerns due to its toxic components. Bioremediation utilizes microbial organisms to metabolism and remove these contaminants. The aim of this study was to enrich a microbial community and examine its potential to degrade petroleum hydrocarbon. Through successive enrichment, we obtained a bacterial consortium using crude oil as sole carbon source. The 16 S rRNA gene analysis illustrated the structural characteristics of this community. Metagenomic analysis revealed the specific microbial organisms involved in the degradation of cyclohexane and all the six BTEX components, with a demonstration of the versatile metabolic pathways involved in these reactions. Results showed that our consortium contained the full range of CDSs that could potentially degrade cyclohexane, benzene, toluene, and (*o*-, *m*-, *p*-) xylene completely. Interestingly, a single taxon that possessed all the genes involved in either the activation or the central intermediates degrading pathway was not detected, except for the *Novosphingobium* which contained all the genes involved in the upper degradation pathway of benzene, indicating the synergistic interactions between different bacterial genera during the hydrocarbon degradation.

**Supplementary Information:**

The online version contains supplementary material available at 10.1186/s13568-023-01541-y.

## Introduction

Petroleum pollution usually resulted from spills and leakages during oil exploration, storage and transportation. This attracts worldwide concern due to the large number of hazardous and toxic constituents in the petroleum (Bierkens and Geerts, 2014). Petroleum hydrocarbons can be classified in four groups based on their solubility: saturated hydrocarbons, aromatic hydrocarbons, resins, and asphaltenes (Han et al. 2018). Benzene, toluene, ethylbenzene, and xylene (BTEX) are volatile simple aromatic hydrocarbons, commonly present in gasoline and other consumer products. One of the characteristics of BTEX is their relatively water-insolubility, they can diffuse rapidly once introduced into aquifers, accounting up to 90% of the dissolved pollutants in groundwater contamination plumes (Suarez and Rifai 2002). Combining with their high mobility in the environment and toxicity to human and animals, BTEX are included in the priority pollutants list by the U.S. Environmental Protection Agency (Keith and Telliard 1979). Therefore, BTEX contamination is of particular concern and efficient remediation strategies are of great demand.

Bioremediation is an cost-effective and eco-friendly approach to remove pollutants from the environment. Many microorganisms are capable to degrade BTEX under aerobic conditions, including *Acidocella* (Eze 2021), *Pseudomonas, Burkholderia, Cupriavidus (*Bacosa et al. 2021), *Streptomyces* (Hocinat et al. 2020), *Acinetobacter* (Zhou et al. 2016), *Comamonas (*Jiang et al. 2015), *Bacillus* (Wongbunmak et al. 2020), *Microbacterium* (Wongbunmak et al. 2017), *Massilia* (Son et al. 2021), *Paraburkholderia* (Lee et al. 2019), *Variovorax* (Benedek et al. 2021), *Rhodococcus* (Orro et al. 2015). The stability of the aromatic hydrocarbon is the biggest common challenge for organisms to acquire and utilize. Under aerobic condition, the oxygenases initiate the oxidation of aromatic ring and transform them into several key central intermediates via upper pathways (Harayama et al. 1992; Lipscomb 2008). These central intermediates, mainly catechol, protocatechuate, gentisate (2,5-dihydroxybenzoate) or homogentisate (2,5-dihydroxyphenylacetate), are “activated” for ring cleavage and then converted into intermediary metabolites such as acetyl-CoA, succinyl-CoA and pyruvate via central pathways (Fuchs et al., 2011).

The majority of studies performed in the field of BTEX biodegradation are focused on the isolation, cultivation, and characterization of microorganisms. While, pure cultures of single isolates are powerless when multiple contaminants appear in the environment. It has been reported that microbial consortium is more efficient on BTEX degradation than single microorganisms (Mukherjee and Bordoloi 2012), and the development of microbial consortia for BTEX remediation has attracted increasing attention. The indigenous microorganisms present in the polluted environments are more competitive than exogenous microorganisms because they have adapted to the polluted environmental conditions (Deng et al. 2017). Hence, it is an effective strategy to cultivate hydrocarbon-degrading bacterial consortia from the oil-polluted site for the remediation of organic pollutants.

Karamay oilfield is the first large oilfield discovered in China in 1955. It is still one of the largest oilfields in China with a yearly output over ten million tons of oil. In this study, an indigenous bacterial consortium, derived from the crude oil polluted soil in Karamay oilfield, was isolated and investigated using metagenomics. Our main purpose is to cultivate an hydrocarbon-degrading consortium and identify microorganisms playing a role in BTEX degradation. Our research assigned specific pathways to specific microorganisms in cyclohexane and BTEX pathways, which will enhance our understanding of hydrocarbon-degrading microorganisms and expand their application in petroleum contamination bioremediation.

## Materials and methods

### Soil sampling

The crude oil-polluted samples were collected from the Karamay oilfield located in the Xinjiang Uygur Autonomous Region, China. Topsoil samples were acquired aseptically, placed in sterilized sealable polythene bags and transported to the laboratory on ice. The samples were later filtered through a 2 mm pore size sieve and stored at -80℃ for microbial analysis.

### Enrichment cultures and growth conditions

Approximately 1 g of the crude oil-polluted soil was added to Erlenmeyer flasks (250 mL) containing 50 mL of culture medium composed of KH_2_PO_4_ (5.7 g/L), K_2_HPO_4_•3H_2_O (3.0 g/L), NaCl (0.5 g/L), NH_4_Cl (5.67 g/L), and MgSO_4_•7H_2_O (2.24 g/L). 10 g/L of sterile-filtered crude oil was added to the flask as the sole carbon and energy source. The cultures were grown at 30℃ with shaking at 150 rpm and maintained within a 15-day subculture. After six subculture, 30 mL aliquots were centrifuged for 10 min at 4000×g.

### DNA extraction

Total genomic DNA were extracted using the E.Z.N.A.® Soil DNA Kit (Omega Bio-tek, Norcross, GA, U.S.). The concentration and purity of DNA extracts were determined with TBS-380 and NanoDrop2000, respectively. The quality of DNA extracts was checked on 1% agarose gel. DNA from the soil samples and enrichment culture were applied to metagenomic analysis.

### Sequencing of bacterial 16 S rRNA genes

Bacterial 16 S rRNA gene (V3-V4 region) were amplified using the forward primer 338 F (5’-ACTCCTACGGGAGGCAGCAG-3’) and the reverse primer 806R(5’-GGACTACHVGGGTWTCTAAT-3’) by an ABI GeneAmp® 9700 PCR thermocycler (ABI, CA, USA). The PCR reaction (20 µL final volume) contained 4 µL of 5 × *TransStart* FastPfu buffer, 2 µL of 2.5 mM dNTPs, 0.8 µL of each primer (5 µM), 0.4 µL of *TransStart* FastPfu DNA Polymerase, and 10 ng of the extracted DNA as the template. The PCR amplification was performed as follows: initial denaturation at 95 ℃ for 3 min, 27 cycles of denaturing at 95 ℃ for 30 s, annealing at 55 ℃ for 30 s, followed by extension at 72 ℃ for 45 s. The final extension was carried out at 72 ℃ for 10 min. The PCR products were purified from 2% agarose gel using the AxyPrep DNA Gel Extraction Kit (Axygen Biosciences, Union City, CA, USA), and quantified using Quantus™ Fluorometer (Promega, USA). Purified amplicons were pooled in equimolar and paired-end sequenced on an Illumina MiSeq PE300 platform platform (Illumina, San Diego,USA) according to the standard protocols by Majorbio Bio-Pharm Technology Co. Ltd. (Shanghai, China).

### Metagenome sequencing, assembly and analysis

DNA extract was fragmented to an average size of about 400 bp using Covaris M220 (Gene Company Limited, China) for paired-end library construction. Paired-end library was constructed using NEXTFLEX Rapid DNA-Seq (Bioo Scientific, Austin, TX, USA). Adapters containing the full complement of sequencing primer hybridization sites were ligated to the blunt-end of fragments. Paired-end sequencing was performed on Illumina NovaSeq (Illumina Inc., San Diego, CA, USA) at Majorbio Bio-Pharm Technology Co., Ltd. (Shanghai, China). After truncating the barcode and primer sequences, fastp version 0.20.0 (Chen et al. 2018) was used to remove low-quality reads (length < 50 bp or with a quality value < 20 or having N bases). Metagenomics data were assembled using MEGAHIT version 1.1.2 (Li et al. 2015). Contigs with the length being or over 300 bp were selected as the final assembling results. Coding DNA sequences (CDSs) were identified with prodigal (Hyatt et al. 2010). The operational taxonomic units (OTUs) was clustered using CD-HIT version 4.6.1 (Li and Godzik 2006) with the default value of 97%. Non-redundant gene catalog were aligned to NCBI NR database using Diamond version 0.8.35 (Buchfink et al. 2015) for taxonomic annotations. Functional annotation was performed with diamond version 0.8.35 and the KEGG database (Kanehisa and Goto 2000).

### Sequence deposition

Raw reads of the microbiomes 16 S rRNA gene amplicons and the whole-metagenome shotgun sequence of the enrichment consortium have been deposited in the NCBI Sequence Read Archive (SRA) and are available under the BioProject accession number PRJNA892061 and SRA SRP404615, PRJNA895942 and SRA SRP405463.

## Results

### Bacterial diversity of the sampling site and the enrichment culture

**Fig. 1:**
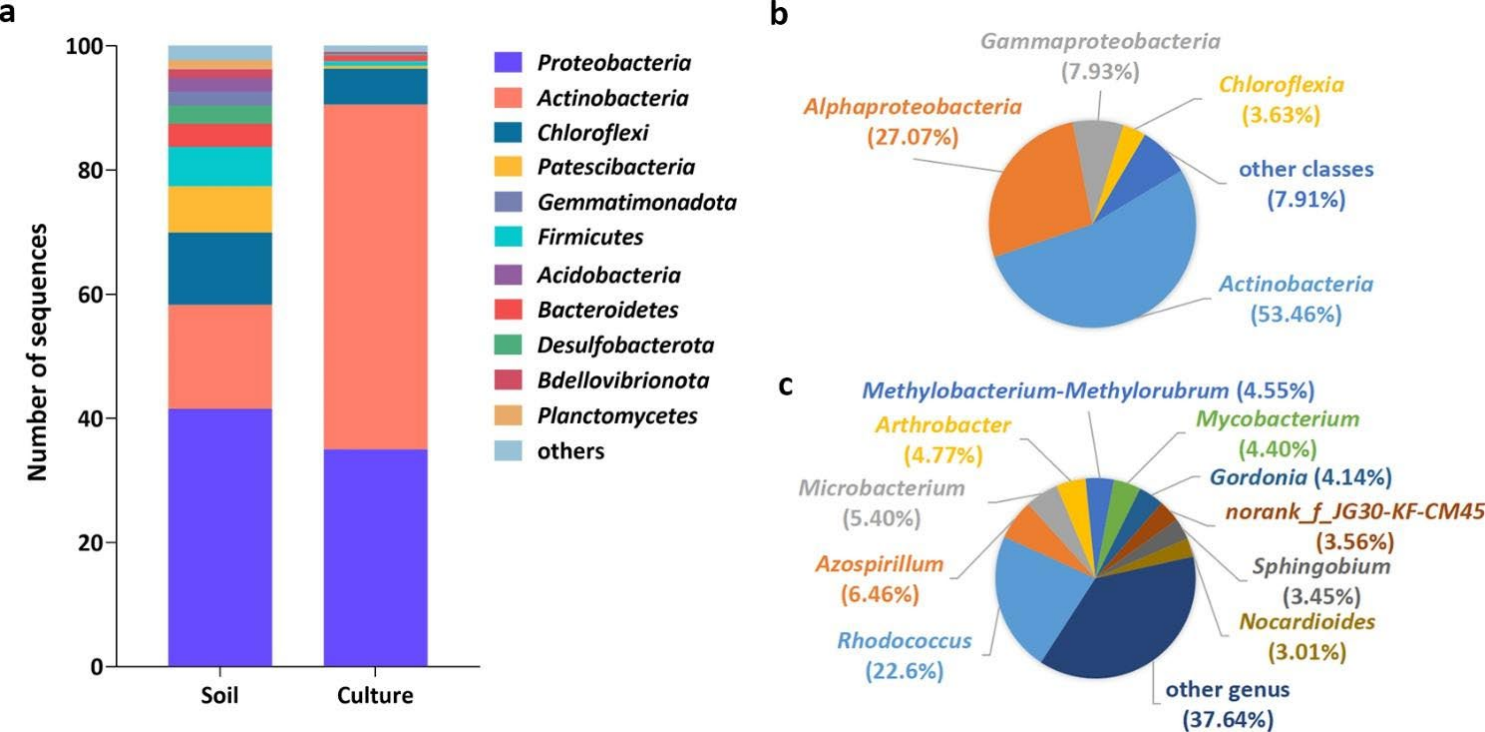
**a** Bacterial community composition of the polluted soil and enrichment culture. Relative abundance of taxonomic classification of the enrichment culture at a **b** class level and **c** genus level

A total of 1914 OTUs were identified from 340,472 sequences for all samples. The relative abundances at the bacterial phyla level showed the dominance of *Proteobacteria* (41.5%) and *Actinobacteria* (16.8%) in the polluted soil samples (Fig. 1a). The enrichment culture was also dominated by *Proteobacteria* and *Actinobacteria*, while the relative abundances were different (35.0% and 55.5%, respectively). At class level, *Actinobacteria* and *Alphaproteobacteria* dominated the enrichment culture, accounting for 53.5% and 27.0%, respectively (Fig. 1b). At genus level, the top ten dominant genera of the enrichment culture were *Rhodococcus*, *Azospirillum, Microbacterium, Arthrobacter, Methylobacterium-Methylorubrum, Mycobacterium, Gordonia, norank_f__JG30-KF-CM45, Sphingobium* and *Nocardioides* (Fig. 1c).

### Identification of aliphatic and aromatic hydrocarbon‑degrading coding DNA sequences

Functional analysis of the metagenome derived from the microbial enrichment culture revealed that 12 potential enzymatic classes represented by 1128 coding DNA sequences (CDSs) were involved in the degradation of aliphatic and aromatic hydrocarbons.

**Fig. 2:**
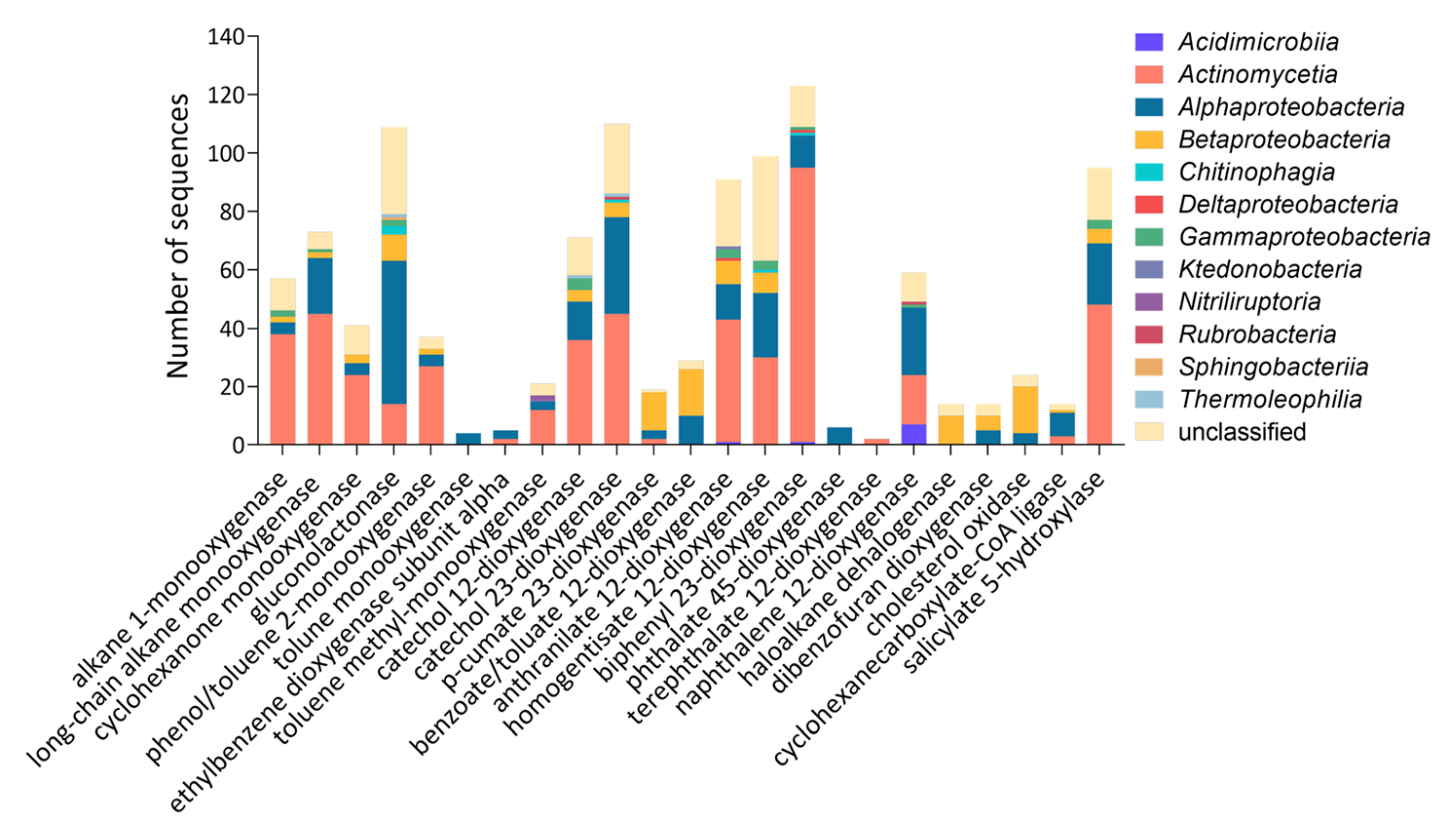
The number of sequences associated with specific hydrocarbon-degrading enzymes of the enrichment culture with a taxonomic classification at a class level

**Fig. 3:**
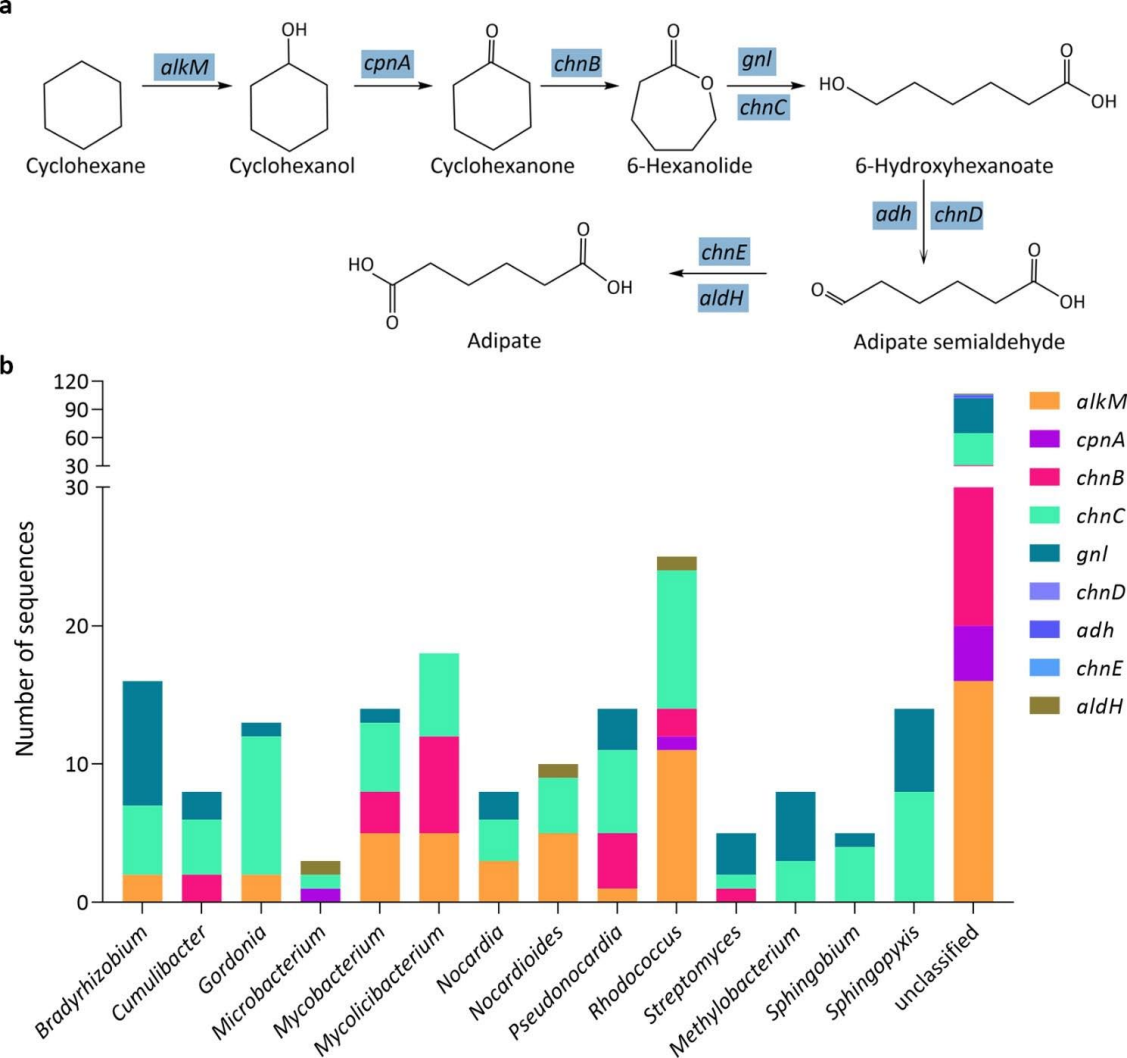
**a** Biodegradation of cyclohexane via the Baeyer-Villiger oxidation pathway, **b** The number of the CDSs involved in this biodegradation pathway at a genus level. Genera that contain over three of the nine genes or over five CDSs are shown

The enzymes considered to be responsible for the degradation of aliphatic hydrocarbons included alkane 1-monooxygenase, long-chain alkane monooxygenase, cyclohexanone monooxygenase and gluconolactonase (Fig. 2). Two hundred and eighty CDSs were detected to play a role in aliphatic hydrocarbon degradation, in which 121 CDSs belonged to the *Actinomycetia* and 76 CDSs to the *Alphaproteobacteria*. It is worth mentioning that our consortium contained all the genes involved in cyclohexane degradation, including the *alkM*, *cpnA*, *chnB*, *chnC*, *gnl*, *chnD adh*, *chnE* and *aldH* genes (Fig. 3a). These genes were assigned to seventy four genera (Table S1, despite of the unclassified genera), and seven of which contain more than 10 CDSs: *Rhodococcus* (25 CDSs), *Mycolicibacterium* (18 CDSs), *Bradyrhizobium* (16 CDSs), *Mycobacterium* (14 CDSs), *Sphingopyxis* (14 CDSs), *Gordonia* (13 CDSs) and *Nocardioides* (10 CDSs) (Fig. 3b).

In aerobic conditions, the first step of aromatic hydrocarbon biodegradation is an oxidation catalyzed by monooxygenases (hydroxylases) or by dioxygenases. In the enrichment culture, five hundred and thirty-seven CDSs were detected as oxygenases. Among those CDSs, one hundred and ten were catechol 2,3-dioxygenase, ninety-nine were homogentisate 1,2-dioxygenase, ninety-five were benzoate/toluate 1,2-dioxygenase, fifty-nine as phthalate 4,5-dioxygenase, thirty-seven as catechol 1,2-dioxygenase, thirty as phenol/toluene 2-monooxygenase, twenty-nine as anthranilate 1,2-dioxygenase, twenty-one as *p*-cumate 2,3-dioxygenase, fourteen as naphthalene 1,2-dioxygenase, fourteen as terephthalate 1,2-dioxygenase, fourteen as toluene monooxygenase, six as biphenyl 2,3-dioxygenase, five as toluene methyl-monooxygenase, four as ethylbenzene dioxygenase (Fig. 2).

### Upper pathway in the degradation of BTEX

The results showed that our consortium contained the genes involved in all the steps for the conversion of benzene to catechol, toluene to either benzoate or 3-methylcatechol, and (*o-, m-, p-*,) xylene to (*o-, m-, p-*,) methylbenzoate.

**Fig. 4:**
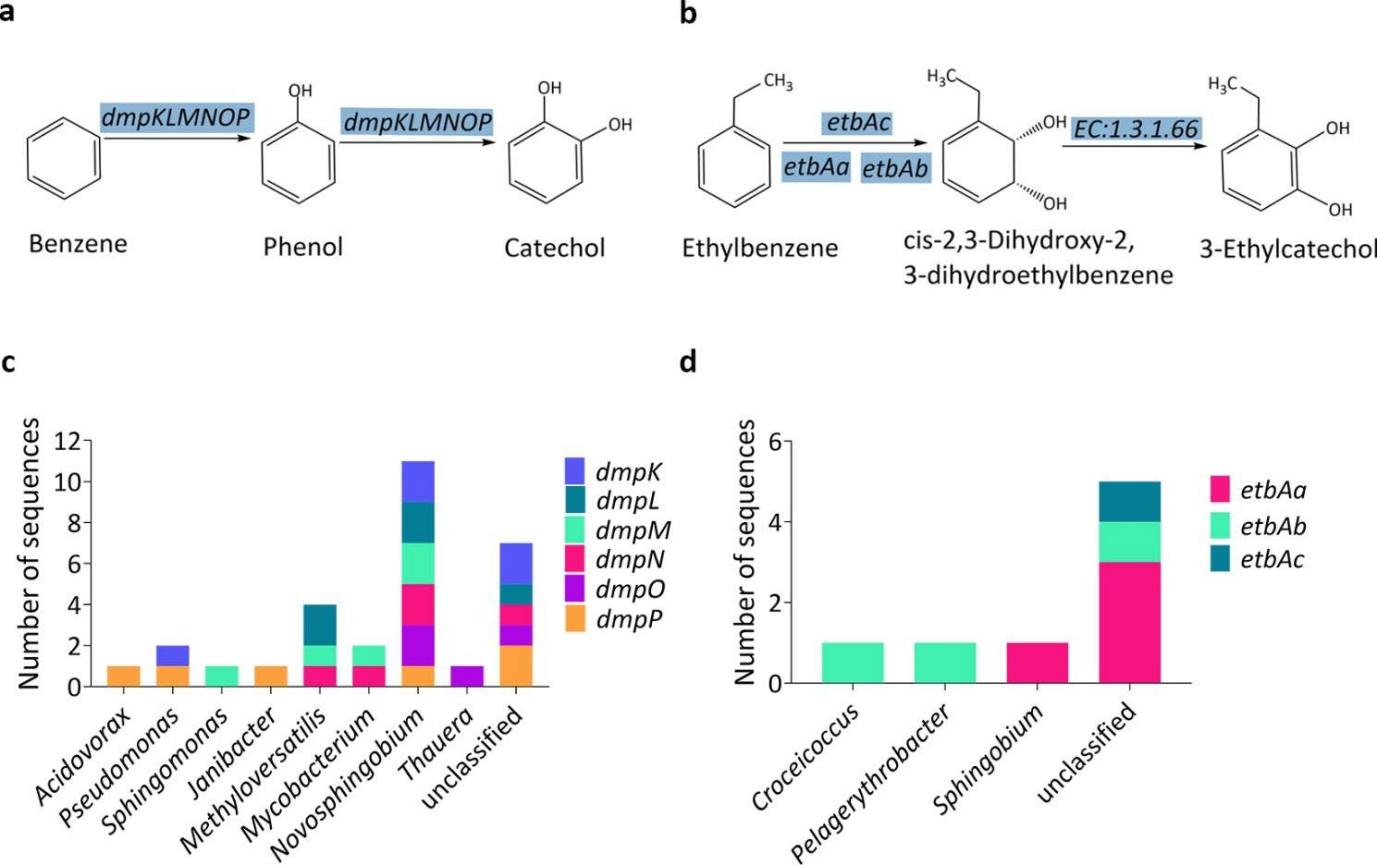
The activation of **a** benzene by monooxygenases and **b** ethylbenzene by dioxygenases. The number of CDSs involved in the **c** benzene and **d** ethylbenzene activation at a genus level

The metagenome results revealed that thirty putative CDSs were involved in the upper pathway in the degradation of benzene. These CDSs were classified as phenol/toluene 2-monooxygenase, corresponding to six genes *dmpKLMNOP* (Fig. 4a). Taxonomic assignments indicated that eleven CDSs affiliated to the bacterial genus *Novosphingobium*, which contained all the six genes. The other twelve CDSs were assigned to bacterial genus *Acidovorax* (1 CDS), *Pseudomonas* (2 CDSs), *Sphingomonas* (1 CDS), *Janibacter* (1 CDS), *Methyloversatilis* (4 CDSs), *Mycobacterium* (2 CDSs) and *Thauera* (1 CDS) (Fig. 4c).

Ethylbenzene degradation is initiated by ethylbenzene dioxygenase (*etbAaAbAc*) and subsequently transformed to 3-ethylcatechol (Fig. 4b). In our consortium, eight CDSs participated in the initial oxidation of ethylbenzene, three of which were assigned to *Croceicoccus*, *Pelagerythrobacter* and *Sphingobium* (Fig. 4d).

**Fig. 5:**
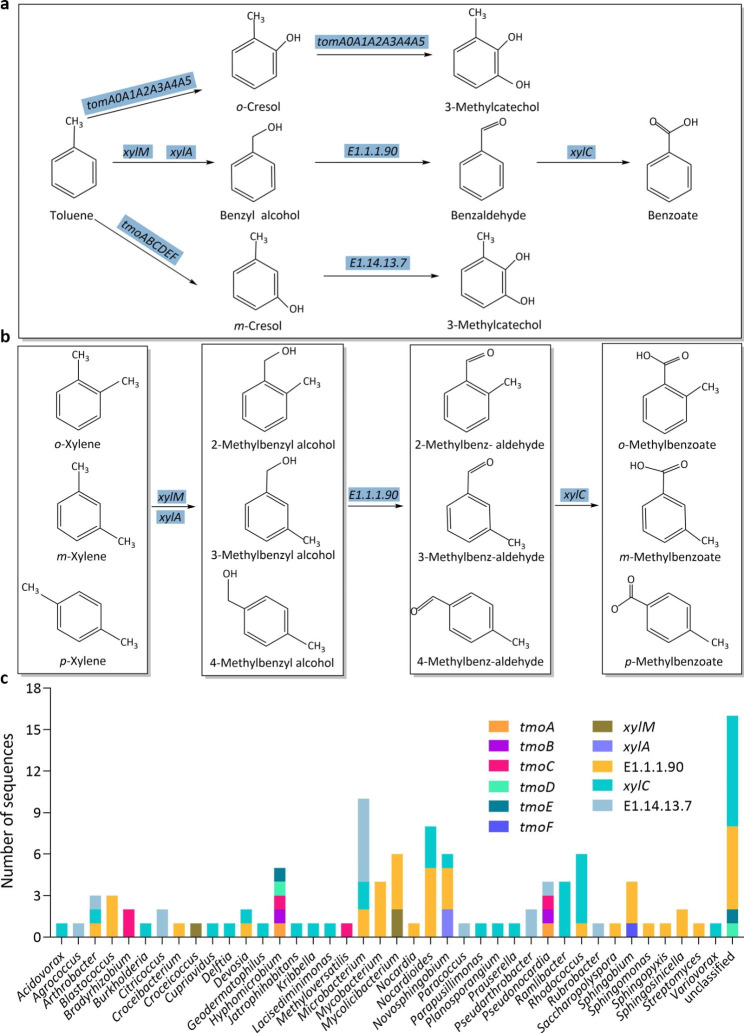
The activation of **a** toluene and **b** xylene by monooxygenases. **c** The number of CDSs involved in the toluene and xylene activation at a genus level. Tulene 2-monooxygenase genes (*tmoA0A1A2A3A4A5*) belong to the same genera as *dmpKLMNOP* genes shown in Fig. 4c

In the toluene degradation pathway of our consortium, the initial oxidation step was catalyzed by toluene 2-monooxygenases (*tomA0A1A2A3A4A5*), toluene monooxygenases (*tmoABCDEF*) or toluene methyl-monooxygenases (*xylMA*), producing *o-*cresol, *m-*cresol or benzyl alcohol, respectively (Fig. 5a). The toluene 2-monooxygenases can further transform the *o-*cresol to 3-methylcatechol, these genes (*tomA0A1A2A3A4A5*) possess the same functions as *dmpKLMNOP* genes and assigned to the same genera shown in Fig. 4c. Genus assignments demonstrated that 8 out of 14 of toluene monooxygenases (*tmoABCDEF*) belonged to *Hyphomicrobium* (5 CDSs) and *Pseudonocardia* (3 CDSs) (Fig. 5c). Meaningwhile, five CDSs that belonged to *Mycolicibacterium* (2 CDSs), *Novosphingobium* (2 CDSs) and *Croceicoccus* (1 CDS), were potentially involved in toluene methyl-monooxygenase (*xylMA*) degradation step. Benzyle alcohol is transformed to benzoate by two dehydrogenases, E1.1.1.90 and XylC, while *m-*cresol is metabolized to 3-methylcatechol by a phenol 2-monooxygenase (E1.14.13.7). Our results indicate that 92 CDSs were identified based on these three genes (*E1.1.1.90*, *xylC* and *E1.14.13.7*), which affiliate to 35 bacterial genera (despite of these unclassified genera), including *Microbacterium* (10 CDSs), *Nocardioides* (8 CDSs), *Rhodococcus* (6 CDSs), *Mycobacterium* (4 CDSs) and *Sphingobium* (4 CDSs), etc.

The degradation pathway of xylene is similar to toluene. Toluene methyl-monooxygenase genes *xylM* and *xylA* initiate the degradation of ortho-, meta-, and para-xylenes (Fig. 5b). The aryl-alcohol dehydrogenase (*E1.1.1.90*) and benzaldehyde dehydrogenase (*xylC*) are involved in the subsequent oxidation of 2-methylbenzyl alcohol, 3-methylbenzyl alcohol and 4-methylbenzyl alcohol to *o*-methylbenzoate, *m*-methylbenzoate and *p*-methylbenzoate.

### Central intermediates degradation pathways of BTEX

**Fig. 6:**
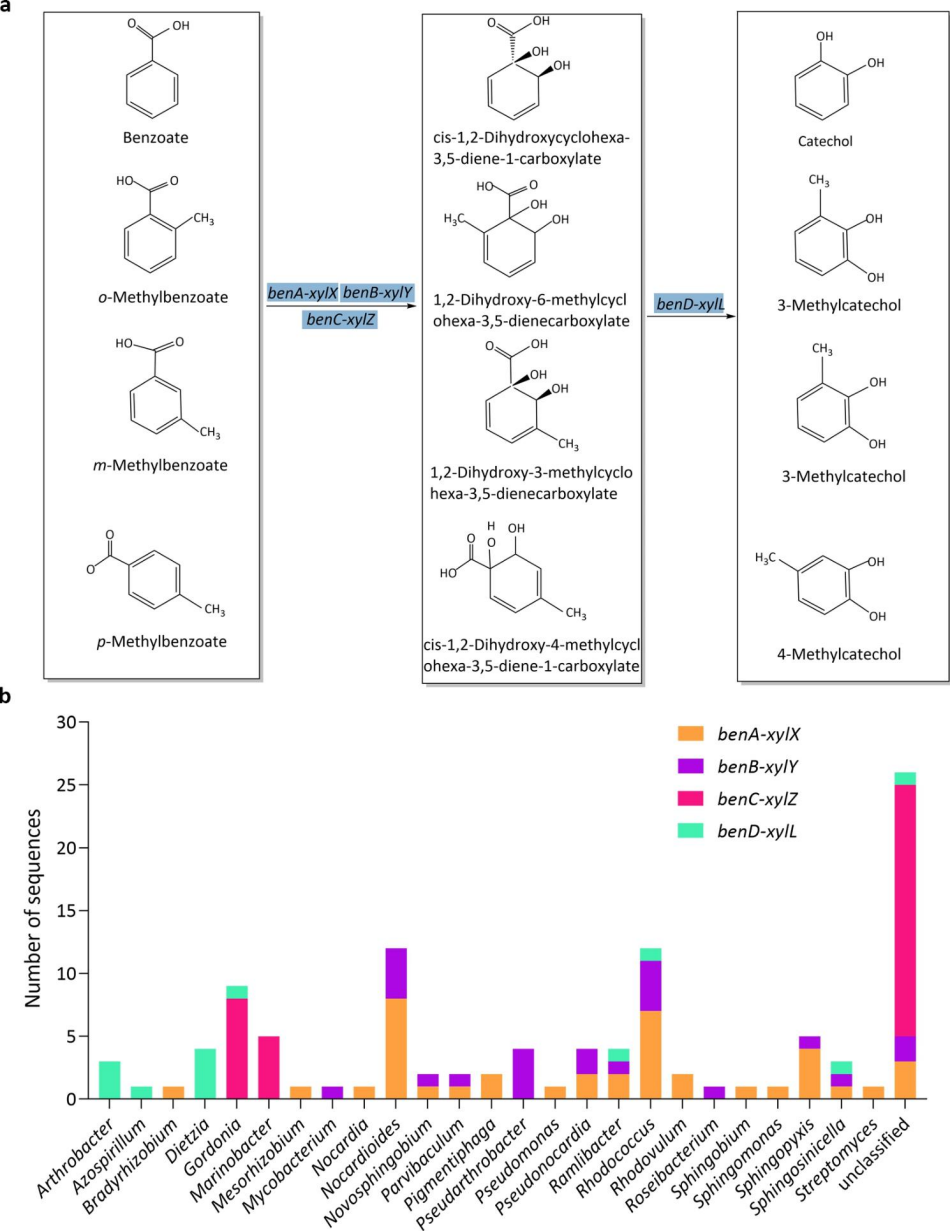
**a** The transformation of benzoate and (*o-, m-, p-*,) methylbenzoate to catechol and (3-, 4-) methylcatechol, respectively. **b** The number of CDSs involved in the degrading pathways at a genus level

The degradation of toluene and xylene produces benzoate and methylbenzoate, which are further transformed by the benzoate/toluate 1,2-dioxygenase (*benA-xylX*, *benB-xylY* and *benC-xylZ*) and dihydroxycyclohexadiene carboxylate dehydrogenase (*benD-xylL*) to catechol and methylcatechol (Fig. 6a). A total of 109 CDSs played a role in the benzoate degradation pathway, which were assigned to 25 bacterial genera (despite of these unclassified genera). The majority of these CDSs were assigned to *Nocardioides* (12 CDSs), *Rhodococcus* (12 CDSs), *Gordonia* (9 CDSs), *Marinobacter* (5 CDSs), and *Sphingopyxis* (5 CDSs) (Fig. 6b).

**Fig. 7:**
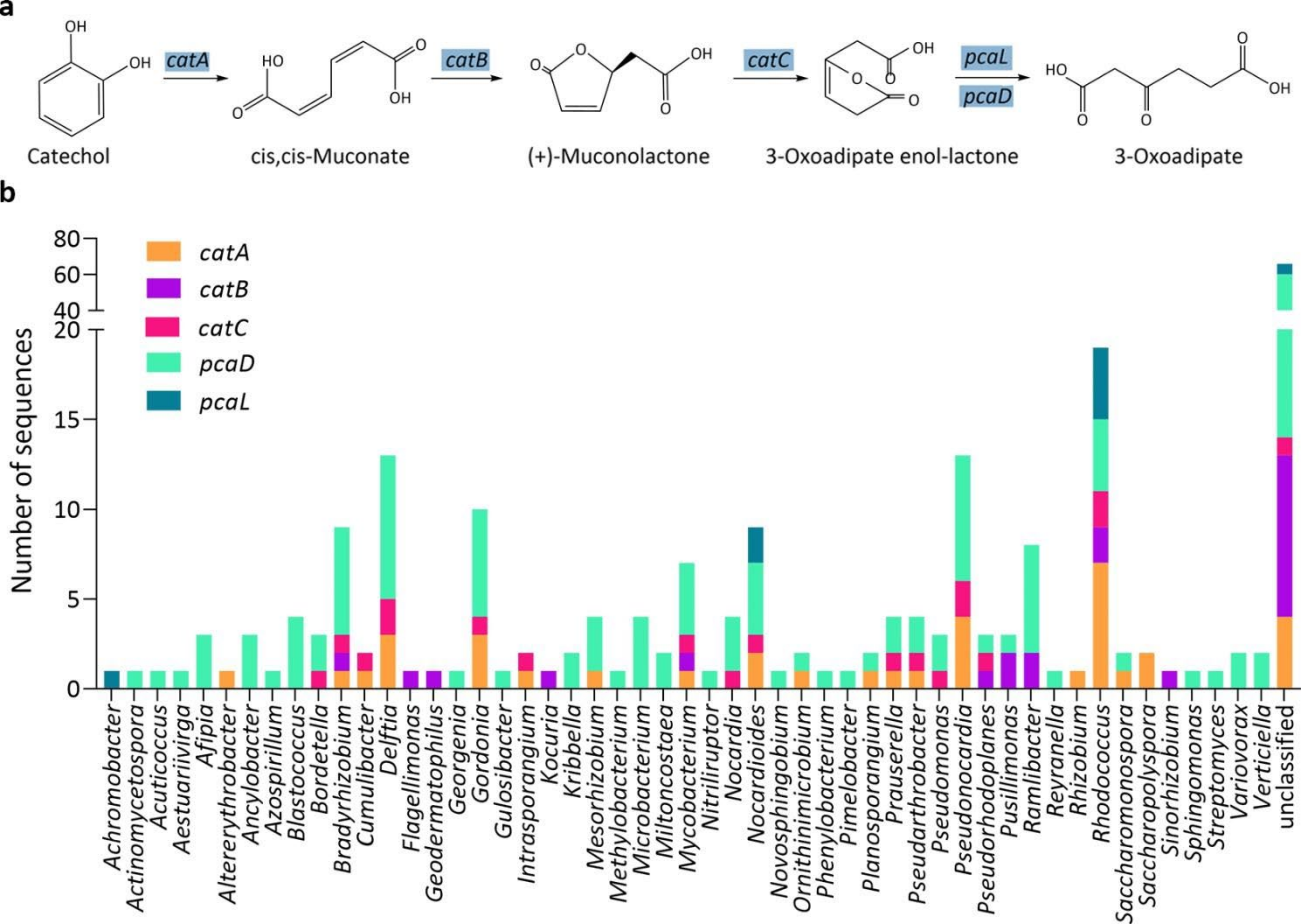
**a** The ortho-cleavage of catechol. **b** The number of CDSs involved in the ortho-cleavage of catechol at a genus level

In the ortho-cleavage of catechol pathway, catechol is first oxidized to cis,cis-muconate by catechol 1,2-dioxygenase (*catA*), then converted to 3-oxoadipate enol-lactone by muconate cycloisomerase (*catB*) and muconolactone *D*-isomerase (*catC*), and further metabolized to 3-oxoadipate with 3-oxoadipate enol-lactonases (*pcaDL*) (Fig. 7a). The metagenome data showed that our consortium contained 237 CDSs involved in this pathway, of which, 19 belonged to *Rhodococcus*, 13 to *Delftia*, 13 to *Pseudonocardia*, 10 to *Gordonia*, 9 to *Bradyrhizobium*, 9 to *Nocardioides*, 8 to *Ramlibacter*, and 7 to *Mycobacterium* (Fig. 7b). Our results demonstrated that 66 CDSs could not be assigned to any specific genus, and the other 83 CDSs were affiliated to 43 bacterial genera.

**Fig. 8:**
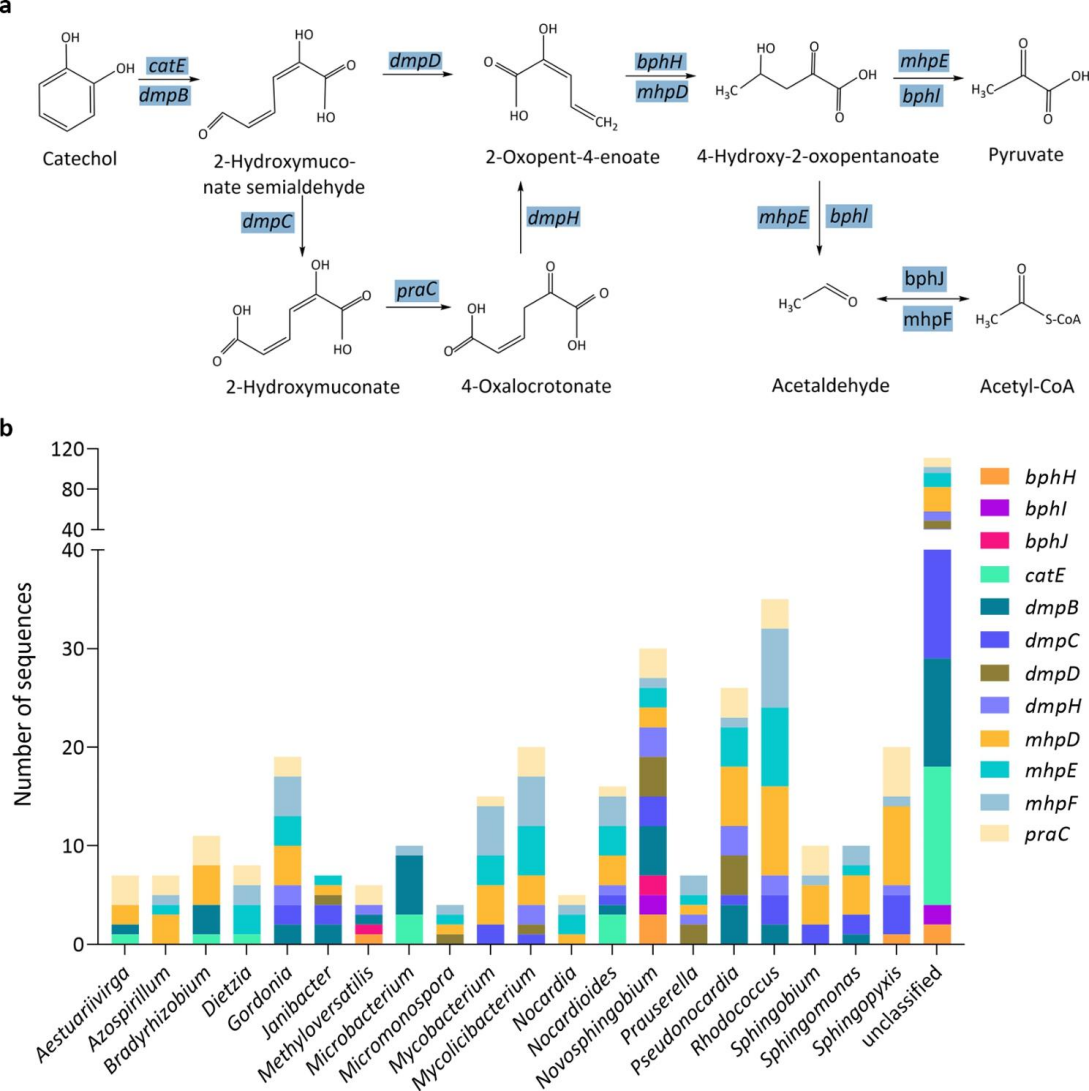
**a** The meta-cleavage of catechol. **b** The number of CDSs involved in the meta-cleavage of catechol at a genus level. Genera that contain over four of the twelve genes are shown

In the meta-cleavage of catechol pathway, the catechol and methylcatechol are initiated by the catechol 2,3-dioxygenase, then converted to a 4-hydroxy-2-oxoacid intermediate, which is cleaved by the aldolase to produce pyruvate, acetaldehyde or propanal. The propanal and acetaldehyde are transformed by the aldehyde dehydrogenase to propanoyl-CoA and acyl-CoA, respectively (Fig. 8a). Our results showed that the consortium contained all the genes involved in these reactions, including the *catE*, *todF*, *dmpBCDH*, *mhpDEF*, *bphHIJ* and *praC* genes, adding up to 523 CDSs. The *todF* gene, involved in the transformation of 3-methylcatechol to 2-hydroxy-2,4-pentadienoate, corresponding to only 1 CDS affiliated to a unclassified genus. Four hundred and eleven of these 523 CDSs affiliated to 100 specific bacterial genera, and the other 112 CDSs were assigned as unclassified genera (Table S2). Among the 100 bacterial genera, 19 of which contained more than four genes, including *Rhodococcus* (35 CDSs), *Novosphingobium* (30 CDSs), *Pseudonocardia* (26 CDSs), *Mycolicibacterium* (20 CDSs), *Sphingopyxis* (20 CDSs), *Gordonia* (19 CDSs), *Nocardioides* (16 CDSs), *Mycobacterium* (15 CDSs), *Bradyrhizobium* (11 CDSs), *Sphingobium* (10 CDSs), *Sphingomonas* (10 CDSs), *Dietzia* (8 CDSs), *Aestuariivirga* (7 CDSs), *Azospirillum* (7 CDSs), *Janibacter* (7 CDSs), *Prauserella* (7 CDSs), *Methyloversatilis* (6 CDSs), *Nocardia* (5 CDSs) and *Micromonospora* (4 CDSs) (Fig. 8b).

The activation of ethylbenzene degradation resulted in the production of 3-ethylcatechol. The central metabolism of 3-ethylbenzene is initiated with a ring cleavage reaction by the 2,3-dihydroxyethylbenzene 1,2-dioxygenase (e*tbC*). The product 2-hydroxy-6-oxo-octa-2,4-dienoate is then transformed to 2-hydroxy-2,4-pentadienoate by the hydrolase gene *etbD*. The *etbC* gene is not detected in our consortium, and the *etbD* gene corresponded to only 1 CDS, which assigned to the genus *Sphingobium*.

## Discussion

Crude oil contamination is of great concern owning to the toxic components that are devastating to natural habitats or harmful to human and animal lives (Harvey et al. 2012;Garr et al., 2014;McKee and White, 2014). The restoration of petroleum hydrocarbon contaminated soil usually requires multiple microorganisms due to the complexity of the pollutant. The successive enrichment using crude oil resulted in a bacterial consortium that has the potential to degrade aliphatic and aromatic hydrocarbon. The *n-*alkanes are the main parts of petroleum hydrocarbon and can be utilized as sole carbon source by bacteria. The biodegradation of *n-*alkanes is initiated by the alkane hydroxylases, including alkanes 1-monooxygenase (AlkB or AlkB-like) and long-chain alkane monooxygenase (LadA) (Li et al. 2008, 2020; Rojo 2009). A total of 130 CDSs were identified as alkane hydroxylases, mainly affiliated to Actinomycetia (83 CDSs) and Alphaproteobacteria(23 CDSs) (Fig. 2).

Cyclohexane and its derivatives are also the main contents of petroleum hydrocarbon. The cyclohexane can be transformed to *ε*-caprolactone by oxygenases and dehydrogenase (Tiralerdpanich et al. 2018; Dallinger et al. 2016). Then this lactone is split by an caprolactone hydrolase to yield 6-hydroxyhexanoic acid, which further oxidized to adipate (Dallinger et al. 2016). These enzymes include cyclopentanol dehydrogenase (CpnA), cyclohexanone monooxygenase (ChnB), gluconolactonase (Gnl), epsilon-lactone hydrolase (ChnC), alcohol dehydrogenase (Adh), 6-hydroxyhexanoate dehydrogenase (ChnD), aldehyde dehydrogenase (AldH) and 6-oxohexanoate dehydrogenase (ChnE) (Fig. 3a). A total of 314 CDSs were detected in our consortium, indicating the significant potential of cyclohexane degradation ability of the microbial community (Fig. 3b).

The degradation of benzene, toluene, ethylbenzene and (*o-*, *m-* and *p-*) xylene is initiated by progressive oxidation to produce benzoate or catechol. The degradation pathways of BTEX can be diverse, for instance, more than five toluene degrading pathways have been discovered, including the dioxygenase mediated pathway, toluene 2-monooxygenase, toluene 3-monooxygenase, toluene 4-monooxygenase mediated pathway and TOL pathways (Parales et al., 2008). The metagenome of our consortium contains genes that code for phenol/toluene 2-monooxygenase (*dmpKLMNOP*), ethylbenzene dioxygenase (*etbAaAbAc*), toluene monooxygenase (*tmoABCDEF*), toluene 2-monooxygenases (*tomA0A1A2A3A4A5*) and toluene methyl-monooxygenase (*xylAM*) (Figs. 4a and b and 5a and b). These genes, *dmpKLMNOP, etbAaAbAc, tmoABCDEF, tomA0A1A2A3A4A5, xylAM*, have been reported to be responsible for the activation of BTEX degradation (Dalvi et al. 2012; Jindrová et al. 2002). The presence of monooxygenases in our metagenome data indicates that our consortium can potentially activate BTEX compounds mainly through monooxygenase pathway. The central intermediates, benzoate and catechol, are then transformed to substrates of the citrate cycle. Metagenome results showed that our consortium contained all the genes involved in the central intermediates degradation pathways, including benzoate degradation (Fig. 6a), catechol ortho-cleavage (Fig. 7a) and catechol meta-cleavage (Fig. 8a). Many researchers also enriched BTEX degrading consortia, they sometimes lack a number of enzymes to completely metabolize the BTEX (Eze 2021). While, our consortium contains a full range of genes involved in the activation pathways of all the six BTEX components and the central intermediates metabolism pathways of benzene, toluene and (*o*-, *m*-, *p*-) xylene.

The functional genes involved in the BTEX degradation are initially studied in *Rhodococcus* and *Pseudomonas*, such as the *todE* in *Pseudomonas putida* F1 (Zylstra and Gibson 1991; Busch et al. 2010), *tmoA* in *Pseudomonas mendocina* KR1 (Kukor and Olsen 1991), *xylMA* in *Pseudomonas putida* mt-2 (Greated et al. 2002), *akbD* in *Rhodococcus* sp. DK17 (Kim et al. 2004), *catA/*C12O and C23O in *Pseudomonas putida* ND6 (Jiang et al. 2004), *bnzA1* in *Rhodococcus opacus* B4 (Na et al. 2005), and *dmpL* in *Pseudomonas putida* CF600 (van der Meer 1997;Suenaga et al. 2009). Since then, many other genera have been proved to be able to degrade BTEX. In this study, taxonomic annotation revealed that the CDSs involved in these reactions belong to diverse genera. The majority of genera responsible for the activation of BTEX were *Novosphingobium, Microbacterium*, *Mycolicibacterium, Nocardioides, Hyphomicrobium* and *Pseudonocardia.* More genera played a role in the degradation of intermediates, including *Rhodococcus*, *Novosphingobium*, *Pseudonocardia*, *Mycolicibacterium*, *Sphingopyxis, Gordonia*, *Nocardioides*, *Mycobacterium*, *Bradyrhizobium*, *Sphingobium*, *Sphingomonas*, *Dietzia*, *Aestuariivirga*, *Azospirillum*, *Janibacter*, *Prauserella*, *Methyloversatilis*, *Nocardia* and *Micromonospora*. Metagenomic analysis showed distinctive degrading microorganisms in different bacterial consortia. The bacterial consortium enriched by Eze et al. (Eze 2021) could active BTEX degradation through both the monooxygenase and dioxygenase pathways, and the *Acidocella* and *Aquabacter* have the highest potential for the BTEX degradation. Bacterium consortium EC20, could degrade BTEX through TOL pathway and TOD pathway, is dominated by *Pseudomonas*, *Mesorhizobium*, *Achromobacter*, *Stenotrophomonas*, and *Halomonas* (Deng et al. 2017). The *Geobacter*-related bacteria were enriched from the center of the BTEX contaminated plume (Farkas et al. 2017). The differentiation of degrading genera among different studies might be attribute to the diverse sources and enrichment strategies of bacterial consortia. Metagenomic analyses of our study illustrated the diversity of genera and the corresponding genes involved in the BTEX degradation, which would provide theoretical basis for their potential application in the BTEX bioremediation.

The enrichment of bacterial consortium for hydrocarbon degradation were mainly dominated by *Pseudomonas* (Tiralerdpanich et al. 2018; Oba et al. 2014), while our consortium is dominated by *Rhodococcus.* The genus of *Rhodococcus* can degrade a wide variety of organic and xenobiotic compounds, including aliphatic and aromatic hydrocarbons (Duetz et al. 2001; Yakimov et al. 1999). The metabolic pathways of *Rhodococcus* have been extensively studied in microbial biotechnology fields worldwide (Kim et al. 2018). The biodegradation potential of different *Rhodococcus* species has been evaluated based on genome analyses and “-omics” approaches (Zampolli et al. 2019), such as the degradation of BTEX by *R.opacus* R7 (Orro et al. 2015)d *jostii* DK17 (Yoo et al. 2012), naphthalene by *R. opacus* M213 (Pathak et al. 2016). In our consortium, the genus of *Rhodococcus* was involved in the degradation of *n-*alkanes, cyclohexane and aromatic hydrocarbons. Interestingly, the *Rhodococcus* did not contain the full range of genes involved in either degradation pathway. This indicates that *Rhodococcus* might have synergistic interactions with different bacterial genera during the hydrocarbon degradation.

## Electronic supplementary material

Below is the link to the electronic supplementary material.

Supplementary Material 1

## Data Availability

Raw sequence data has been deposited in the NCBI Sequence Read Archive (SRA) and are available under BioProject number PRJNA892061 and PRJNA895942.

## Declarations

**Ethics approval and consent to participate**.

**Consent for publication**.
