# Supplementary material for: Metagenomic analysis reveals specific BTEX degrading microorganisms of a bacterial consortium
Source: AMB Express. 2023 May 17;13:48. doi: 10.1186/s13568-023-01541-y (PMC10192505; doi:10.1186/s13568-023-01541-y)
Supplement: Supplementary file 1 — Supplementary Material 1 [file 13568_2023_1541_MOESM1_ESM.docx]

**Supplementary information**

Table S1 Genus assignment of genes involved in the cyclohexane degradation pathway of the enrichment culture.

| Genus level classification | *alkM* | *cpnA* | *chnB* | *chnC* | *gnl* | *adh* | *aldH* | *chnD* | *chnE* |
| --- | --- | --- | --- | --- | --- | --- | --- | --- | --- |
| *Acidovorax* | 2 |  |  |  |  |  |  |  |  |
| *Actinomarinicola* |  |  |  | 1 |  |  |  |  |  |
| *Acuticoccus* |  |  |  | 2 | 3 |  |  |  |  |
| *Aeromicrobium* |  |  |  | 1 |  |  |  |  |  |
| *Aestuariivirga* |  |  |  |  | 3 |  |  |  |  |
| *Afipia* |  |  | 1 |  | 2 |  |  |  |  |
| *Aggregatilinea* |  |  |  |  |  | 1 |  |  |  |
| *Agromyces* |  |  | 2 | 2 |  |  |  |  |  |
| *Alcanivorax* | 1 |  |  |  |  |  |  |  |  |
| *Amycolatopsis* | 1 |  |  | 1 |  |  |  |  |  |
| *Ancylobacter* |  |  |  | 1 |  |  |  |  |  |
| *Antricoccus* |  |  | 1 |  |  |  |  |  |  |
| *Aquirhabdus* |  |  |  | 1 |  |  |  |  |  |
| *Arthrobacter* |  |  | 1 |  |  |  | 2 |  |  |
| *Azospirillum* |  |  |  |  | 1 |  |  |  |  |
| *Blastococcus* |  |  |  | 3 |  |  |  |  |  |
| *Blastomonas* | 1 |  |  |  |  |  |  |  |  |
| ***Bradyrhizobium*** | 2 |  |  | 5 | 9 |  |  |  |  |
| *Calidifontimicrobium* |  |  |  |  | 1 |  |  |  |  |
| *Candidimonas* |  |  |  |  | 1 |  |  |  |  |
| *Clostridioides* |  |  |  | 1 |  |  |  |  |  |
| ***Cumulibacter*** |  |  | 2 | 4 | 2 |  |  |  |  |
| *Delftia* |  |  |  |  | 1 |  |  |  |  |
| *Devosia* |  |  |  |  | 2 |  |  |  |  |
| *Diaphorobacter* |  |  | 1 |  |  |  |  |  |  |
| *Dietzia* | 1 |  |  | 3 |  |  |  |  |  |
| *Ensifer* |  |  |  |  | 1 |  |  |  |  |
| *Extensimonas* |  |  |  | 1 |  |  |  |  |  |
| *Flavisolibacter* |  |  |  |  | 1 |  |  |  |  |
| *Geodermatophilus* |  |  | 1 |  |  |  |  |  |  |
| ***Gordonia*** | 2 |  |  | 10 | 1 |  |  |  |  |
| *Hoeflea* |  |  |  |  | 1 |  |  |  |  |
| *Iamia* |  |  |  | 2 |  |  |  |  |  |
| *Janibacter* |  |  |  | 1 |  |  |  |  |  |
| *Ketobacter* |  |  |  | 1 |  |  |  |  |  |
| *Kibdelosporangium* |  |  |  | 1 |  |  |  |  |  |
| *Marinobacter* | 1 |  |  | 1 |  |  |  |  |  |
| *Mesorhizobium* |  |  |  | 1 | 1 |  |  |  |  |
| ***Methylobacterium*** |  |  |  | 3 | 5 |  |  |  |  |
| ***Microbacterium*** |  | 1 |  | 1 |  |  | 1 |  |  |
| *Microbispora* |  |  |  | 1 |  |  |  |  |  |
| *Mucilaginibacter* |  |  |  |  | 1 |  |  |  |  |
| ***Mycobacterium*** | 5 |  | 3 | 5 | 1 |  |  |  |  |
| *Mycobacteroides* |  |  |  | 3 |  |  |  |  |  |
| ***Mycolicibacterium*** | 5 |  | 7 | 6 |  |  |  |  |  |
| ***Nocardia*** | 3 |  |  | 3 | 2 |  |  |  |  |
| ***Nocardioides*** | 5 |  |  | 4 |  |  | 1 |  |  |
| *Nordella* |  |  |  |  | 1 |  |  |  |  |
| *Novosphingobium* |  |  |  | 3 |  |  |  |  |  |
| *Ornithinicoccus* |  |  |  |  |  |  | 2 |  |  |
| *Parvibaculum* |  |  | 2 | 2 |  |  |  |  |  |
| *Phenylobacterium* |  |  |  |  | 2 |  |  |  |  |
| *Pigmentiphaga* |  |  | 1 |  |  |  |  |  |  |
| *Pimelobacter* |  |  |  |  |  |  | 1 |  |  |
| *Prauserella* |  |  |  |  |  |  | 2 |  |  |
| *Pseudarthrobacter* |  |  |  |  | 2 |  | 2 |  |  |
| *Pseudomonas* |  |  |  |  | 2 |  |  |  |  |
| ***Pseudonocardia*** | 1 |  | 4 | 6 | 3 |  |  |  |  |
| *Pseudorhodoplanes* |  |  |  |  | 1 |  |  |  |  |
| *Quisquiliibacterium* |  |  |  |  | 2 |  |  |  |  |
| *Ralstonia* |  |  |  | 1 |  |  |  |  |  |
| *Ramlibacter* |  |  |  | 1 | 1 |  |  |  |  |
| *Rhodobacter* |  |  |  | 1 |  |  |  |  |  |
| ***Rhodococcus*** | 11 | 1 | 2 | 10 |  |  | 1 |  |  |
| *Roseomonas* |  |  |  | 1 | 1 |  |  |  |  |
| *Sinorhizobium* |  |  |  |  | 3 |  |  |  |  |
| ***Sphingobium*** |  |  |  | 4 | 1 |  |  |  |  |
| *Sphingomonas* |  |  |  |  | 1 |  |  |  |  |
| ***Sphingopyxis*** |  |  |  | 8 | 6 |  |  |  |  |
| *Streptomonospora* |  |  |  |  |  |  | 1 |  |  |
| ***Streptomyces*** |  |  | 1 | 1 | 3 |  |  |  |  |
| *Variovorax* |  |  |  |  | 1 |  |  |  |  |
| *Xanthobacter* |  | 1 |  |  | 2 |  |  |  |  |
| *Xylophilus* |  | 1 |  |  |  |  |  |  |  |
| **unclassified** | 16 | 4 | 11 | 34 | 37 | 2 | 1 | 1 | 1 |

Genera in bold are those contain over three of the nine genes or over five CDSs shown in figure 3b.

Table S2 Genus assignment of genes involved in the cetachol meta-cleavage pathway of the enrichment culture.

| Genus level classification | *bphH* | *bphI* | *bphJ* | *catE* | *dmpB* | *dmpC* | *dmpD* | *dmpH* | *mhpD* | *mhpE* | *mhpF* | *praC* | *todF* |
| --- | --- | --- | --- | --- | --- | --- | --- | --- | --- | --- | --- | --- | --- |
| *Acidovorax* | 1 |  |  |  |  |  |  |  | 4 |  |  | 2 |  |
| ***Aestuariivirga*** |  |  |  | 1 | 1 |  |  |  | 2 |  |  | 3 |  |
| *Afipia* |  |  |  | 2 |  |  |  |  | 1 |  |  | 5 |  |
| *Agrococcus* |  |  |  |  | 1 |  |  |  |  |  |  |  |  |
| *Alicyclobacillus* |  |  |  |  |  |  |  |  |  |  |  | 1 |  |
| *Amycolatopsis* |  |  |  |  |  |  |  |  |  |  | 1 |  |  |
| *Ancylobacter* |  |  |  | 1 |  |  |  |  |  |  |  | 1 |  |
| *Ancylobacter* |  |  |  |  |  |  |  |  |  |  |  |  |  |
| *Antricoccus* | 1 |  |  |  |  |  |  |  |  |  |  |  |  |
| *Aquabacterium* |  |  |  |  |  |  | 1 |  |  |  |  |  |  |
| *Arthrobacter* |  |  |  | 1 | 1 |  |  |  | 1 |  |  |  |  |
| *Aurantiacibacter* |  |  |  |  |  |  | 1 |  |  |  | 1 |  |  |
| ***Azospirillum*** |  |  |  |  |  |  |  |  | 3 | 1 | 1 | 2 |  |
| *Blastococcus* |  |  |  | 1 |  |  |  |  |  |  |  |  |  |
| ***Bradyrhizobium*** |  |  |  | 1 | 3 |  |  |  | 4 |  |  | 3 |  |
| *Carbonactinospora* |  |  |  |  |  |  |  |  | 1 |  |  |  |  |
| *Chelativorans* |  |  |  |  | 1 |  |  |  |  |  |  |  |  |
| *Citricoccus* |  |  |  |  | 1 |  |  |  |  |  |  |  |  |
| *Comamonas* |  |  |  |  |  |  |  |  |  | 1 |  |  |  |
| *Croceicoccus* |  |  |  |  | 1 |  |  |  |  |  |  |  |  |
| *Cucumibacter* |  |  |  |  | 1 |  |  |  | 2 |  |  |  |  |
| *Cupriavidus* |  |  |  |  |  |  |  |  |  |  | 1 |  |  |
| *Delftia* |  |  |  |  |  |  |  |  | 1 |  |  |  |  |
| *Devosia* |  |  |  | 2 |  |  |  |  |  |  |  | 1 |  |
| ***Dietzia*** |  |  |  | 1 |  |  |  |  |  | 3 | 2 | 2 |  |
| *Duganella* |  |  |  | 1 |  |  |  |  |  |  |  |  |  |
| *Elioraea* |  |  |  |  |  |  |  | 1 |  |  |  |  |  |
| *Enhydrobacter* |  |  |  |  |  |  |  |  | 1 |  |  |  |  |
| *Enterovirga* |  |  |  | 1 |  |  |  |  |  |  |  |  |  |
| *Escherichia* |  |  |  |  |  |  |  |  |  |  | 1 |  |  |
| *Euzebya* |  |  |  |  |  |  |  |  | 2 | 2 | 2 |  |  |
| *Flavisolibacter* |  |  |  | 1 |  |  |  |  |  |  |  |  |  |
| *Frankia* |  |  |  | 1 |  |  |  |  |  | 1 |  |  |  |
| *Gaiella* |  |  |  | 1 |  |  |  |  |  |  |  |  |  |
| *Geodermatophilus* |  |  |  |  |  |  |  |  | 1 |  | 1 |  |  |
| *Georgenia* |  |  |  | 2 |  |  |  |  |  |  |  |  |  |
| ***Gordonia*** |  |  |  |  | 2 | 2 |  | 2 | 4 | 3 | 4 | 2 |  |
| *Homoserinibacter* |  |  |  | 1 |  |  |  |  |  |  |  |  |  |
| *Hydrogenophaga* |  |  |  |  |  | 1 | 2 |  |  |  |  |  |  |
| *Hyphomicrobium* |  |  |  | 1 |  |  |  |  |  |  |  |  |  |
| ***Janibacter*** |  |  |  |  | 2 | 2 | 1 |  | 1 | 1 |  |  |  |
| *Kocuria* |  |  |  |  | 1 |  |  |  |  |  |  |  |  |
| *Marinobacter* |  |  |  |  |  |  |  |  |  | 1 |  | 1 |  |
| *Marmoricola* |  |  |  |  |  |  |  |  |  |  | 1 |  |  |
| *Mesorhizobium* |  |  |  | 2 | 2 |  |  |  | 2 |  |  | 1 |  |
| *Methylocapsa* |  |  |  |  |  |  |  |  |  |  |  | 1 |  |
| *Methylophilus* |  |  |  |  |  |  |  |  |  |  |  | 1 |  |
| *Methylorubrum* |  |  |  |  |  |  |  |  |  |  |  | 1 |  |
| ***Methyloversatilis*** | 1 |  | 1 |  | 1 |  |  | 1 |  |  |  | 2 |  |
| ***Microbacterium*** |  |  |  | 3 | 6 |  |  |  |  |  | 1 |  |  |
| *Micrococcus* |  |  |  |  | 1 |  |  |  |  |  |  |  |  |
| ***Micromonospora*** |  |  |  |  |  |  | 1 |  | 1 | 1 | 1 |  |  |
| *Microterricola* |  |  |  | 1 |  |  |  |  |  |  |  |  |  |
| *Miltoncostaea* |  |  |  | 1 |  |  |  |  |  |  |  |  |  |
| ***Mycobacterium*** |  |  |  |  |  | 2 |  |  | 4 | 3 | 5 | 1 |  |
| *Mycobacteroides* |  |  |  |  |  |  |  |  |  | 2 | 1 |  |  |
| ***Mycolicibacterium*** |  |  |  |  |  | 1 | 1 | 2 | 3 | 5 | 5 | 3 |  |
| ***Nocardia*** |  |  |  |  |  |  |  |  | 1 | 2 | 1 | 1 |  |
| ***Nocardioides*** |  |  |  | 3 | 1 | 1 |  | 1 | 3 | 3 | 3 | 1 |  |
| *Nonomuraea* |  |  |  |  |  |  |  |  | 1 |  |  |  |  |
| *Nostoc* |  |  |  |  |  | 1 |  |  |  |  |  |  |  |
| *Noviherbaspirillum* | 1 |  |  |  |  |  |  | 2 | 1 |  |  |  |  |
| ***Novosphingobium*** | 3 | 2 | 2 |  | 5 | 3 | 4 | 3 | 2 | 2 | 1 | 3 |  |
| *Ornithinimicrobium* |  |  |  | 1 |  |  |  |  |  |  |  |  |  |
| *Paracoccus* |  |  |  | 1 |  |  |  |  |  |  |  |  |  |
| *Pararobbsia* |  |  |  |  |  |  |  |  |  | 1 |  |  |  |
| *Parvibaculum* |  |  |  |  |  | 1 |  |  |  |  |  |  |  |
| *Pelagerythrobacter* |  |  |  |  |  |  |  |  | 1 |  |  |  |  |
| *Phenylobacterium* |  |  |  |  |  |  |  |  |  |  |  | 1 |  |
| *Phycicoccus* |  |  |  |  |  | 1 |  |  |  |  |  |  |  |
| *Pigmentiphaga* |  |  |  |  |  |  |  |  |  |  |  | 1 |  |
| *Pimelobacter* |  |  |  |  |  |  |  | 1 |  |  |  |  |  |
| ***Prauserella*** |  |  |  |  |  |  | 2 | 1 | 1 | 1 | 2 |  |  |
| *Protaetiibacter* |  |  |  |  |  |  |  |  |  |  | 1 |  |  |
| *Pseudarthrobacter* |  |  |  | 1 | 3 |  |  |  | 2 |  |  |  |  |
| *Pseudolysinimonas* |  |  |  | 3 |  |  |  |  |  |  |  |  |  |
| *Pseudomonas* |  |  |  |  |  | 1 | 1 |  |  |  |  |  |  |
| ***Pseudonocardia*** |  |  |  |  | 4 | 1 | 4 | 3 | 6 | 4 | 1 | 3 |  |
| *Pseudorhizobium* |  |  |  | 1 |  |  |  |  |  |  |  |  |  |
| *Pseudorhodoplanes* |  |  |  | 1 | 1 |  |  |  |  |  |  |  |  |
| *Quisquiliibacterium* |  |  |  |  |  |  |  |  | 1 |  |  |  |  |
| *Ramlibacter* |  |  |  |  |  |  |  |  |  |  |  | 1 |  |
| *Reyranella* |  |  |  |  |  |  |  |  | 3 |  |  |  |  |
| ***Rhodococcus*** |  |  |  |  | 2 | 3 |  | 2 | 9 | 8 | 8 | 3 |  |
| *Rubrivivax* |  |  | 1 |  |  |  |  |  |  |  |  |  |  |
| *Ruegeria* |  |  |  |  | 1 |  |  |  |  |  |  |  |  |
| *Salinibacterium* |  |  |  |  |  |  |  |  |  |  |  | 1 |  |
| *Sinorhizobium* |  |  |  | 1 |  |  |  |  |  |  |  |  |  |
| ***Sphingobium*** |  |  |  |  |  | 2 |  |  | 4 |  | 1 | 3 |  |
| ***Sphingomonas*** |  |  |  |  | 1 | 2 |  |  | 4 | 1 | 2 |  |  |
| ***Sphingopyxis*** | 1 |  |  |  |  | 4 |  | 1 | 8 |  | 1 | 5 |  |
| *Sphingosinicella* | 1 | 1 |  |  |  |  |  |  |  |  |  |  |  |
| *Streptomyces* |  |  |  |  |  |  | 1 |  |  | 1 |  |  |  |
| *Tardibacter* |  |  |  |  |  |  |  |  | 1 |  |  |  |  |
| *Tepidimonas* |  |  |  |  | 1 |  |  |  |  |  |  |  |  |
| *Thauera* |  |  |  |  | 2 | 1 |  |  |  |  |  | 1 |  |
| *Tianweitania* |  |  |  |  |  |  |  |  |  |  |  | 1 |  |
| *Tsuneonella* |  |  | 1 |  |  |  |  |  |  |  |  |  |  |
| *Variovorax* |  |  |  |  |  |  | 1 |  |  |  |  |  |  |
| *Zhengella* |  |  |  |  | 1 |  |  |  |  |  |  |  |  |
| **unclassified** | 2 | 2 |  | 14 | 11 | 12 | 8 | 9 | 24 | 14 | 6 | 9 | 1 |

Genera in bold are those contain over four of the twelve genes shown in figure 8b.
